# Antiandrogen-Equipped Histone Deacetylase Inhibitors Selectively Inhibit Androgen Receptor (AR) and AR-Splice Variant (AR-SV) in Castration-Resistant Prostate Cancer (CRPC)

**DOI:** 10.3390/cancers15061769

**Published:** 2023-03-15

**Authors:** Balaji Chandrasekaran, Subhasish Tapadar, Bocheng Wu, Uttara Saran, Ashish Tyagi, Alexis Johnston, David A. Gaul, Adegboyega K. Oyelere, Chendil Damodaran

**Affiliations:** 1 Rangel School of Pharmacy, Texas A&M University, College Station, TX 77845, USA; 2 Parker H. Petit Institute for Bioengineering & Biosciences, School of Chemistry and Biochemistry, Georgia Institute of Technology, 315 Ferst Dr. NW, Atlanta, GA 30332, USA

**Keywords:** androgen receptor, splice variant, castrate resistant prostate cancer, small molecule, AR-V7

## Abstract

**Simple Summary:**

SBI-46, an antiandrogen-equipped histone deacetylase inhibitor, is the lead compound developed for targeting castration-resistant prostate cancer (CRPC). We determined the anticancer effect of SBI-46 on in vitro and in vivo models of CRPC. In our results, SBI-46 downregulates AR and AR-splice variant (SV) expression and its downstream target genes in CRPC cells. Additionally, SBI-46 inhibits AR expression and nuclear localization in the presence of DHT. We further demonstrated that SBI-46 induces apoptosis by activating the pro-apoptotic genes Bax, cleaved PARP, and cleaved caspase-9, as well as downregulating the expression of the antiapoptotic genes Bcl2 and BCl-xL. Additionally, the oral administration of SBI-46 abrogates the growth of C4-2B and 22Rv1 CRPC xenograft tumors that express AR or both AR and AR-SV. Finally, our results demonstrate that SBI-46 exerts an anticancer effect by inhibiting AR and AR-SV in preclinical models of CRPC.

**Abstract:**

Background: Epigenetic modification influences androgen receptor (AR) activation, often resulting in prostate cancer (PCa) development and progression. Silencing histone-modifying enzymes (histone deacetylases-HDACs) either genetically or pharmacologically suppresses PCa proliferation in preclinical models of PCa; however, results from clinical studies were not encouraging. Similarly, PCa patients eventually become resistant to androgen ablation therapy (ADT). Our goal is to develop dual-acting small molecules comprising antiandrogen and HDAC-inhibiting moieties that may overcome the resistance of ADT and effectively suppress the growth of castration-resistant prostate cancer (CRPC). Methods: Several rationally designed antiandrogen-equipped HDAC inhibitors (HDACi) were synthesized, and their efficacy on CRPC growth was examined both in vitro and in vivo. Results: While screening our newly developed small molecules, we observed that SBI-46 significantly inhibited the proliferation of AR^+^ CRPC cells but not AR^-^ CRPC and normal immortalized prostate epithelial cells (RWPE1) or normal kidney cells (HEK-293 and VERO). Molecular analysis confirmed that SBI-46 downregulated the expressions of both AR^+^ and AR-splice variants (AR-SVs) in CRPC cells. Further studies revealed the downregulation of AR downstream (PSA) events in CRPC cells. The oral administration of SBI-46 abrogated the growth of C4-2B and 22Rv1 CRPC xenograft tumors that express AR or both AR and AR-SV in xenotransplanted nude mice models. Further, immunohistochemical analysis confirmed that SBI-46 inhibits AR signaling in xenografted tumor tissues. Conclusion: These results demonstrate that SBI-46 is a potent agent that inhibits preclinical models of CRPC by downregulating the expressions of both AR and AR-SV. Furthermore, these results suggest that SBI-46 may be a potent compound for treating CRPC.

## 1. Introduction

Castration-resistant prostate cancer (CRPC) is an advanced type of prostate cancer (PCa) that progresses even with low androgen levels [1]. Androgen deprivation therapy (ADT) was the standard first-line treatment for men with advanced prostate cancer or CRPC. Unfortunately, most patients eventually resisted ADT, leading to mortality [2]. Splice variants of the androgen receptor (AR) play a pivotal role in the progression to CRPC and resistance to ADT [2,3].

Several AR variants have been reported in CRPC, among which, splice variant 7 (AR-V7) has been frequently linked to resistance for ADT [4]. Most splice variants lack the C-terminal ligand-binding domain (LBD) but retain the transcriptionally active N-terminal domain (NTD) and DNA-binding domain (DBD), which continuously drive AR signaling even with low androgen levels in CRPC [5]. Enzalutamide is an effective second-generation AR inhibitor that binds to the AR’s LBD, attenuates AR translocation into the nucleus, and impedes the transcriptional activation of androgen-responsive target genes [6]. Several clinical studies have shown that enzalutamide improves the survival of PCa patients in a short window [7]; eventually, patients will develop resistance to enzalutamide [8]. Therefore, it has been postulated that circulating tumor cells express AR-V7, which could be associated with enzalutamide resistance in CRPC [9,10]. Hence, developing novel therapeutic molecules is imperative for the treatment of CRPC.

Recent studies have suggested that epigenetic events could regulate AR signaling [11]. More specifically, histone deacetylase (HDAC) enzymes regulate AR stability and the AR-mediated transcriptional activation of several genes [12,13]. Hence, HDAC inhibitors (HDACi) have been considered a viable therapeutic option for CRPC. To support the notion, several preclinical studies validated that the inhibition of HDACs suppresses the proliferation of PCa by degrading AR expression, resulting in the abrogation of AR signaling [12,14,15]. Clinical studies showed HDACi have beneficial effects on hematological malignancies, but the results are not encouraging in solid tumors such as PCa [16] due to a lack of adequate tumor exposure and dose-limiting toxicities [17].

We have described first-in-class HDAC-inhibiting compounds targeted to prostate tumors by equipping them with the additional ability to bind the androgen receptor (AR). These compounds elicited the signatures of the inhibition of HDACs and AR signaling, and the cohort was selectively more toxic to hormone-dependent (AR^+^) LNCaP than a hormone-independent (AR^−^) DU145. In addition, the antiproliferative activities of these dual-acting compounds are significantly enhanced relative to the separate template compounds [18]. This strategy combines two main-stream therapeutic agents, antiandrogen (enzalutamide) and HDACi templates, to selectively deliver a novel class of bifunctional agents to PCa.

**Figure 1:**
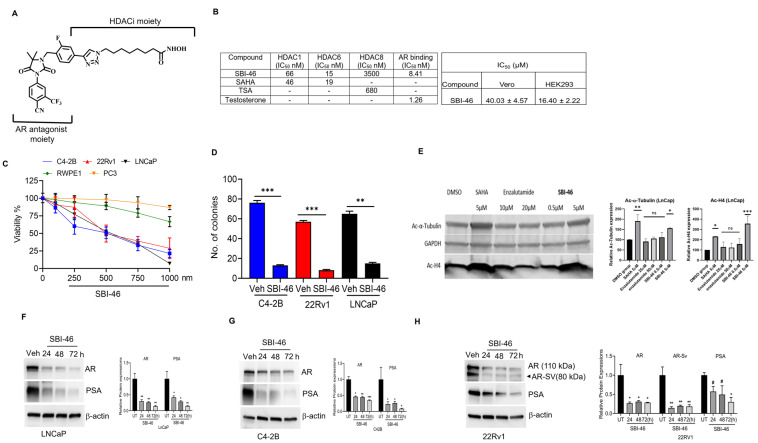
SBI-46 demonstrates on-target effects and inhibits CRPC cell proliferation, growth, and AR and AR-SV expressions. (**A**) Structure of SBI-46. (**B**) HDAC inhibition activities and AR binding affinity of SBI-46. SAHA and TSA were used as controls for HDAC inhibition, while testosterone was used as a control for AR binding and effect of SBI-46 on HEK-293 and VERO cells. (**C**) Effects of SBI-46 treatments (24 h) on the proliferation of CRPC (LNCaP, C4-2B, and 22Rv1) PC3 and RWPE-1 cells. (**D**) LNCaP, C4-2B, and 22Rv1 were treated with SBI-46, and a soft agar colony formation assay was performed. Colonies were counted, and results are presented as means ± SD of experiments performed in triplicate. One-way ANOVA with multiple comparison tests was used to calculate the statistical significance between different experimental groups. ** *p* < 0.01 and *** *p* < 0.001. (**E**) Western blotting analysis of SBI-46 treated or vehicle-treated LNCaP lysates revealed that SBI-46, analogously to SAHA, upregulates the levels of acetylated tubulin and H4. Quantification bars show mean plus standard deviation; ordinary one-way ANOVA compared with the control group, * *p* < 0.0332; ** *p* < 0.0021; *** *p* < 0.0002). (**F**–**H**) Western blotting analysis of SBI-46-treated or vehicle-treated PCa lysates for AR, AR-SV, and PSA expression. Values, mean ± SD. * *p* < 0.05 and ** *p* < 0.01. The uncropped blots are shown in the Supplemental Materials. #, not significant.

In the pursuit of an extensive structure–activity relationship (SAR) study, we have rationally designed and synthesized several antiandrogen-equipped HDAC inhibitors (HDACi) (a detailed structure-activity relationship (SAR) study will be published in a separate manuscript). While screening our newly developed small molecules, we discovered a novel compound, SBI-46 (Figure 1A), which strongly bound to AR and potently inhibited HDACs and the proliferation of AR^+^ CRPC, but not AR- CRPC and healthy immortalized prostate epithelial and kidney cells. SBI-46 inhibited HDACs and AR intracellularly and downregulated AR and AR splice variant (AR-SV) expression, resulting in the growth inhibition of CRPC cells. More importantly, the oral administration of SBI-46 abrogated the growth of AR or both AR and AR-SV-expressing xenografted tumors with no discernible evidence of toxicity.

## 2. Material and Methods

### 2.1. HDAC Inhibition and AR-Binding Assays

In vitro HDAC inhibition was performed by the contract research organization (CRO) BPS Bioscience while an AR-binding assay was performed by Eurofins Panlabs. (St. Charles, MO, USA). These assays were performed through contract agreements with these CROs.

### 2.2. Cell Lines and Reagents

Human PCa cells (LNCaP and 22Rv1) and normal prostate epithelial (RWPE-1) and kidney (HEK-293 and VERO) cells were obtained from American Type Cell Culture (ATCC, Manassas, VA, USA). C4-2B was obtained from the ViroMed Laboratories (Minneapolis, MN). The cells were grown on a specified medium previously described [19]. Dihydrotestosterone (DHT) (1 nM) (Cerilliant, TX, USA) studies were performed in phenol red-free RPMI1640 supplemented with 10% charcoal-stripped FBS (Gemini, West Sacramento, CA, USA).

### 2.3. Cell Viability Assay

The effect of the treatments on prostate cancer and normal prostate epithelial cells’ (LNCaP, 22Rv1, C4-2B, PC3, and RWPE1) viability was evaluated using the Alamar blue assay. Cells were seeded into 96-well plates, and after growing to 60–75% confluency, they were treated with vehicle control (DMSO, Sigma Aldrich, St. Louis, MO, USA) or various concentrations of SBI-46 in respective media for 24 h. At the termination of exposure, cells were incubated with 100 μL of 3% Alamar Blue solution in a complete growth medium at 37 °C for 2 h. Absorbance was measured, and data were analyzed following the manufacturer’s instructions (Life Technologies Corporation, Eugene, OR, USA).

### 2.4. Soft Agar Colony Formation Assay

The CytoSelectTM 96-well In Vitro Tumor Sensitivity Assay Kit was used to perform the colony formation assay to detect anchorage-independent growth (Cell Biolabs, Inc., San Diego, CA, USA). LNCaP, C4-2B, and 22Rv1 cells (5 × 10^3^ cells) that had been exposed to either the vehicle or SBI-46 were harvested and used for the assay following the manufacturer’s instructions. Colonies were manually counted after being stained with 0.005% crystal violet after ten days [20].

### 2.5. Apoptosis Assay

As previously described [21], apoptosis was detected using the annexin V-fluorescein isothiocyanate (FITC) versus propidium iodide (PI) assay (FITC Annexin V Apoptosis Detection Kit I, BD Pharminogen).

### 2.6. Immunofluorescence Analysis

The C4-2B and 22Rv1 cells were plated in eight-chamber glass slides and were serum-deprived for 6 h. The cells were then treated either with the vehicle, SBI-46, or dihydrotestosterone (DHT) or in combination for 24 h and were fixed. Then, the cells were incubated with AR and AR-V7 (ab273500:EPR15656-290) antibodies, followed by secondary antibodies to detect the localization and expression. The cells were analyzed using a KEYENCE fluorescence microscope (BZ-X800/BZ-X810).

### 2.7. Western Blot Analysis

To probe H4 and tubulin acetylation status, LNCaP cells were seeded into a 6-well plate at 1 × 10^6^/well in RPMI for 24–48 h until the confluency reached 80%. Then, 5 µM SAHA, 25 and 50 µM Enzalutamide, 0.5 and 5 µM SBI-46 solutions in DMSO were added to separate cell cultures such that the final DMSO level in each culture was 0.1%. Cells were treated for 6 h and scraped out of the well. The cells were washed with cold PBS three times and lysed with RIPA buffer (110 μL) (VWR, VWRVN653—100 mL) containing phosphatase inhibitor (Fisher Thermo, A32957, Waltham, MA, USA) and protease inhibitor (Fisher Thermo, A32955). The cell lysates were vortexed for 15 s, followed by sonication for 90 s. The lysates were then centrifuged at 14,000 rpm for 10 min, and the supernatants were collected. The total protein concentration was determined using a BCA protein assay kit (BioVision, K813-2500, Milpitas, CA, USA). Based on the results from the BCA assay, the lysates were diluted to make an equal protein concentration, and 20 μg of each lysate was loaded to each well of the TGX MIDI 4–20% gel (Biorad, cat. 5671093, Hercules, CA, USA) and ran at 150 V for 70 min. Subsequently, the gel was transferred onto Turbo PDVF membrane (Biorad, 1704273), and after blocking with 5% BSA for 1–2 h, the membrane was incubated overnight with Ac-Tubulin (Santa Cruz, sc-23950, Dallas, TX, USA), Ac-H4 (Santa Cruz, sc-515319), GAPDH (Santa Cruz, sc-32233), AR (Cell signaling, 5153, Danvers, MA, USA), AR-V7 (ab198394) and (ab273500:EPR15656-290) PSA (ab53774), and β-actin (Cell signaling, 5125) antibodies. On the second day, the membrane was washed with TBST for 3 × 5 min. A relevant secondary antibody (Immunoreagents, part. IR2173, Raleigh, NC, USA) was added, and the membrane was incubated with agitation for 1 h. Bands were quantified using the Odyssey CLx and Biorad Image system. The image J software was used for densitometry analysis.

### 2.8. Xenograft Studies

All animals were maintained in germ-free environments, and the study was carried out per IACUC guidelines that the University of Louisville authorized. Athymic nude Balb/c mice (nu/nu) were obtained from The Jackson Laboratory at 6 weeks of age. C4-2B and 22Rv1 cells (1–1.5 × 10^6^, approximately) were injected into separate flanks of the mice (*n* = 6) for subcutaneous tumor xenograft studies using a final volume of 100 μL of phosphate-buffered saline and Matrigel (1:1). Once the tumors reached ~50–100 mm^3^, the mice were randomly divided into two groups; one group received a vehicle and the other groups received SB1-46 (20 mg/kg) orally for 7 days for 4 weeks. Mice were monitored twice weekly, and tumor volumes were measured once a week.

### 2.9. Immunohistochemical Analysis

Tissue specimens from human prostate tumor 22Rv1 and C4-2B xenografts were formalin-fixed and paraffin-embedded; serial sections (4 µm) were subjected to immunohistochemical analysis using antibodies against AR, AR-V7, Ki67 and PSA. After dewaxing, blocking sections were incubated with primary antibody (overnight, 4 °C) and subsequently incubated with relevant goat antirabbit IgG (2 h, room temperature). Color detection was achieved with 3,3′-Diaminobenzidine and counterstained with hematoxylin. Images were captured via light microscopy (40×) using an Olympus BX43 microscope (Olympus America, Center Valley, PA, USA) [22].

### 2.10. Statistical Analysis

GraphPad Prism 8.0 was used to perform all statistical analyses (GraphPad Software, Inc., La Jolla, CA). Unpaired two-tailed Student’s *t*-tests and a one-way ANOVA analysis were performed for two-group and multiple-group comparisons, respectively. A *p*-value < 0.05 was considered statistically significant, and values were presented as mean ± SD.

## 3. Results

### 3.1. SBI-46 Inhibits HDACs and Tightly Binds to AR In Vitro

To investigate the consequence of the covalent linkage of antiandrogen and HDACi moieties on the inhibition potency of either target, we screened SBI-46 for AR binding and HDAC inhibition against HDAC isoforms 1, 6, and 8. AR-binding affinity was measured with a competition assay using [3H]-mibolerone with testosterone as a positive control. HDAC inhibition was measured using a fluorescence-based assay with SAHA and TSA as positive controls. We observed that SBI-46 bound to AR with a single-digit IC_50_ (Figure 1B and Figure S1) and was much tighter than enzalutamide [18]. Additionally, SBI-46 possessed potent anti-HDAC activity against HDACs 1 and 6; the two HDAC isoforms were suggested to be relevant for PCa growth [13,23,24]. SBI-46, however, showed ~4-fold selectivity for HDAC6. Conversely, SBI-46 was much less potent against HDAC8 (Figure 1B and Figure S2). These results revealed that integrating antiandrogen and HDACi moieties within a single molecule (SBI-46) did not abolish their interaction with either target; an enhancement in AR binding was observed.

### 3.2. SBI-46 Selectively Inhibits the Viability of AR+ and AR-SV CRPC Cells

To elucidate SBI-46’s therapeutic potency, we performed cell viability assays on the AR or both AR and AR-SV (C4-2B, 22Rv1, and LNCaP) and AR-null CRPC cell lines (PC-3) treated for 24 h. We observed that SBI-46 inhibited nanomolar concentrations, the growth of AR+ (LNCaP IC_50_: 500 nM), and AR+ splice variants (AR-SV) and CRPC cells (C4-2B-IC_50_: 480 nM and 22Rv1 IC_50_: 560 nM). On the other hand, SBI-46 was not effective against AR-null PC-3 cells (Figure 1C), suggesting that SBI-46 may selectively sensitize AR (C4-2B), including hormone-dependent LNCaP and AR SV (22Rv1) CRPC cells. Interestingly, no significant toxicity in the normal prostate epithelial cell line (RWPE-1) and kidney cell lines (HEK-293 and VERO) was observed at the tested concentrations of CRPC cells (Figure 1B,C). To determine the effect of SBI-46 on the clonogenic property of CRPC cells, we performed a colony-forming assay. The vehicle-treated cells increased the colony number and size. However, SBI-46 treatment significantly (C4-2B (*p* = 0.001), 22Rv1 (*p* = 0.001), and LNCaP (*p* = 0.001)) reduced the colony-forming ability of CRPC cells (Figure 1D).

### 3.3. SBI-46 Inhibits HDACs Intracellularly

To obtain evidence of intracellular HDAC inhibition, we probed for the effect of SBI-46 on the acetylation status of H4 (a nuclear HDAC class I substrate) and α-tubulin (a cytoplasmic HDAC6 substrate) [25,26] in LNCaP cells with Western blot analysis. We observed that SBI-46 caused a dose-dependent upregulation of acetylated H4 and tubulin (Figure 1E). This result strongly demonstrates the intracellular HDAC inhibition activity of SBI-46.

### 3.4. SBI-46 Inhibits AR and AR-SV in CRPC

Next, we investigated if the inhibition of AR signaling contributes to the antiproliferative effects of SBI-46 against AR+ and AR+ SV CRPC cells. In our Western blot analysis, we observed that the expressions of AR-FL (AR-Full length) and splice variant 7 (AR-V7) were downregulated in SBI-46-treated CRPC cells, with the inhibition of PSA expression downstream of the effector of AR (Figure 1F–H). Together, these results suggest that SBI-46 inhibits AR-FL and AR-SV expression in CRPC cells.

**Figure 2:**
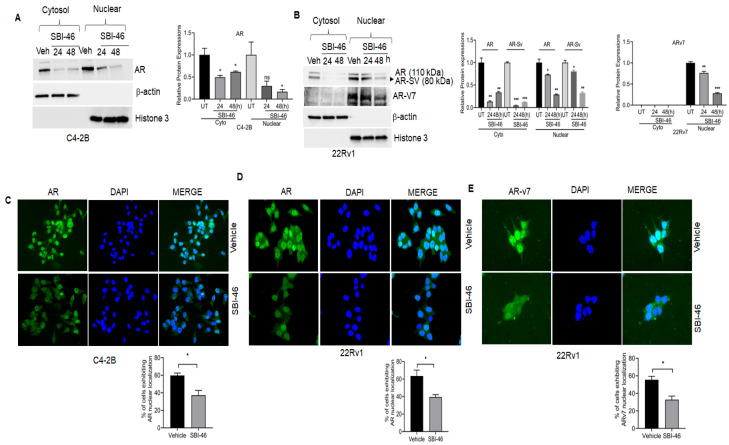
SBI-46 inhibited cytoplasmic and nuclear expressions of AR and AR-V7. (**A**,**B**) Western blot analysis of AR and AR-V7 expression in cytoplasmic and nuclear extracts of C4-2B and 22RV-1 cells after treatment with SBI-46. Densitometry analysis was performed with ImageJ software for the immunoblots. The *p*-values corresponding to various symbols used are: ns, not significant (*p* > 0.05); * *p* ≤ 0.05; ** *p* ≤ 0.01 and *** *p* ≤ 0.001. (**C**–**E**) Representative fluorescent images of AR and ARv7 in C4-2B and 22RV-1 cells 24 h after treatment with vehicle or SBI-46. Quantitative data are presented as mean ± SD. The uncropped blots are shown in the Supplemental Materials.

AR is naturally localized in the cytoplasm. Once it binds to its ligand (such as testosterone), AR translocates into the nucleus and transactivates target genes. However, nuclear-localized AR was seen in CRPC patients. Hence, we examined AR’s cytosolic and nuclear expression in SBI-46-treated 22Rv1 and C4-2B cells. As shown in Figure 2A,B, SBI-46 downregulates both cytoplasmic and nuclear AR and AR-SV expression in C4-2B and 22Rv1 cells. Subsequently, we further confirmed the cyto-nuclear expression of AR by immunofluorescence analysis, and similar results were observed, namely the inhibition of the nuclear expression of AR in SBI-46-treated C4-2B and 22Rv1cells as compared to vehicle-treated cells. (Figure 2C,D). Additionally, the exposure of SBI-46 significantly reduced the nuclear expression of truncated AR variants (AR-V7) in 22Rv1 cells (Figure 2E). To confirm the specificity of the AR-V7 antibody (ab273500), we probed this with PC-3 cells treated with SBI-46, and Western blot analysis was performed it. As expected, the AR-V7 band was not observed (Supplementary Figure S4).

### 3.5. SBI-46 Blocks Nuclear AR Localization in DHT-Treated CRPC Cells

**Figure 3:**
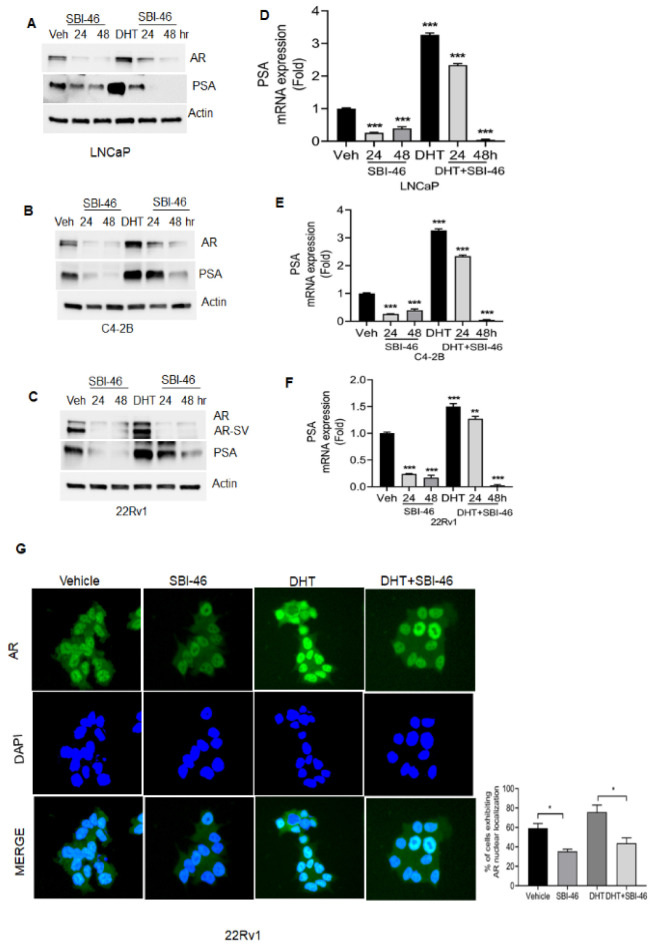
SBI-46 downregulates DHT-induced AR expression in CRPC cells. (**A**–**C**) Western blot and analysis of AR signaling in the vehicle or DHT or combination with SBI-46 in LNCaP, C4-2B, and 22Rv1 cells. (**D**–**F**) Real-time analysis of PSA expression in the vehicle or DHT or combination with SBI-46 in LNCaP, C4-2B, and 22Rv1 cells (**G**). Representative fluorescent images of AR expression in vehicle or DHT or SBI-46-treated 22Rv-1 cells for 24 h. Quantitative data are presented as mean ± SD. Densitometry analysis was performed with ImageJ software for the immunoblots. The *p*-values corresponding to various symbols used are: (*p* > 0.05); * *p* < 0.05; ** *p* < 0.01, and *** *p* < 0.001. The uncropped blots are shown in the Supplemental Materials.

Dihydrotestosterone binds to the AR and elicits the transcriptional activity of AR-regulated genes and functions by inducing proliferation, survival, and differentiation in CRPC [27]. Hence, we challenged either the vehicle, DHT, SBI-46, or a combination in CRPC cells and determined if SBI-46 could override DHT-induced AR signaling. As expected, the treatment of SBI-46 in AR or both AR and AR-SV cells resulted in a marked decrease in the expression of AR and PSA (Figure 3A–C). Furthermore, we confirmed the downregulation of PSA transcripts by real-time PCR (Figure 3D–F).

**Figure 4:**
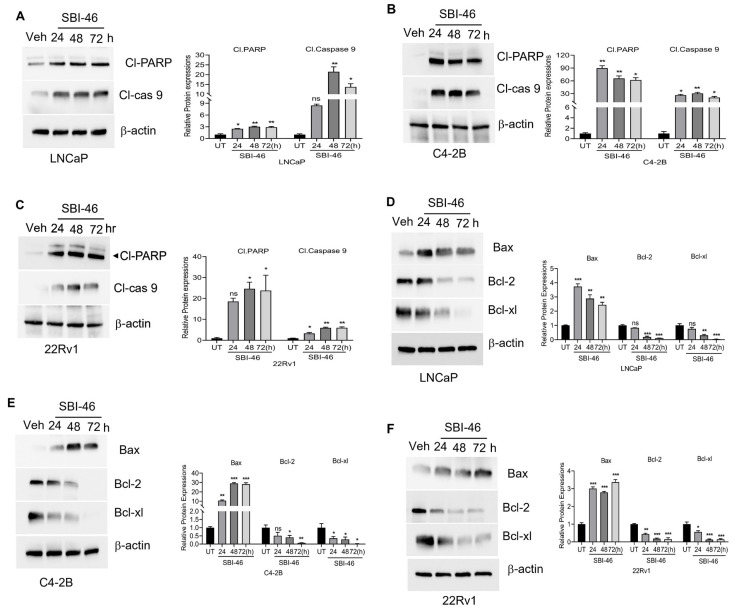
SBI-46 induces apoptosis in PCa cells. (**A**–**F**), Cell lysates from SBI-46-treated PCa cells were analyzed by Western blotting with the indicated antibodies. Densitometry analysis was performed with ImageJ software for the immunoblots. The *p*-values corresponding to various symbols used are: (ns), not significant (*p* > 0.05); * *p* < 0.05; ** *p* < 0.01 and *** *p* < 0.001. The uncropped blots are shown in the Supplemental Materials.

Similarly, DHT-treated 22Rv1 cells showed the increased nuclear localization of AR, while co-treatment with SBI-46 reduced AR’s nuclear localization and expression by immunofluorescence analysis. (Figure 3G). Next, we analyzed whether the inhibition of AR signaling facilitates pro-apoptotic machinery and alters the survival capability of CRPC cells. We examined both pro-apoptotic and survival markers in SBI-46-treated CRPC cells. Figure 4A–F show the downregulation of B-cell lymphoma 2 (Bcl-2) families, Bcl-2 and Bcl-extra-large (Bcl-xL), and the concomitant inductions of pro-apoptotic protein Bax, cleaved Caspase-9, and PARP were seen in SBI-46-treated CRPC cells. The results were further confirmed by annexin V-FITC staining, which revealed the induction of apoptosis in SBI-46-treated C4-2B cells (18% *p* = 0.01) (Figure S3), suggesting that the inhibition of AR signaling indeed facilitates apoptotic machinery in CRPC cells.

### 3.6. SBI-46 Represses Tumor Growth in an In Vivo Xenograft Model of CRPC

**Figure 5:**
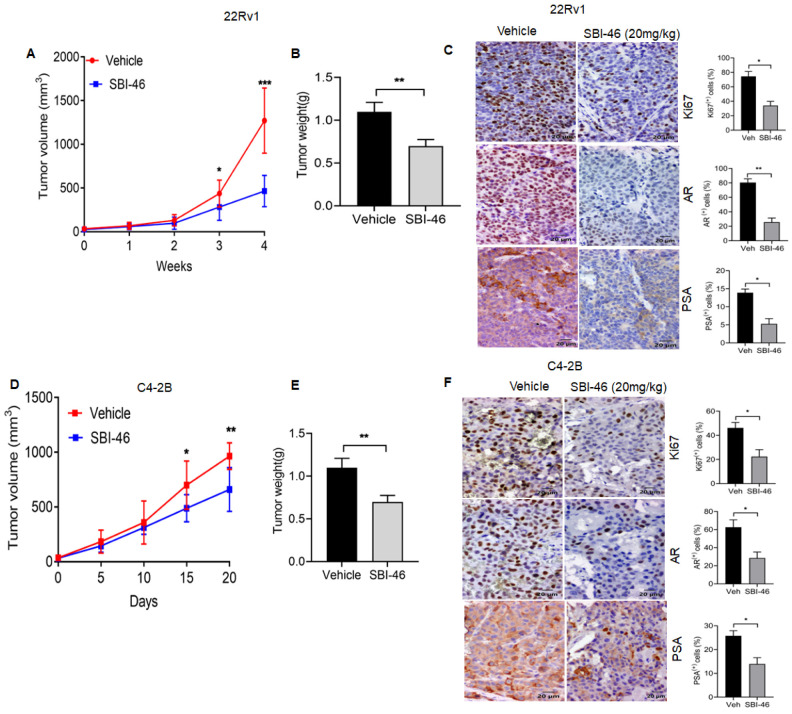
SBI-46 potently suppresses PCa xenografts tumor growth in nude mice. (**A**,**D**) Tumor growth curves for subcutaneous 22Rv1 and C4-2B xenograft nude mice model: nude mice bearing 22Rv1 and C4-2B xenograft tumors (*n* = 6) were treated with either vehicle or SBI-46 (20 mg/kg, Orally), for five days/week for four weeks. (**B**,**E**) Tumor weight of SBI-46- or vehicle-treated 22Rv1 and C4-2B mice xenografts. (**C**,**F**) Representative images of Ki67, AR, and PSA immunostaining were performed in SBI-46- or vehicle-treated mice (22Rv1 and C4-2B) xenografted tumor samples. The *p*-values corresponding to various symbols used are: * *p* < 0.05; ** *p* < 0.01; and *** *p* < 0.001.

Finally, in a nude mice model, we evaluated the efficacy of orally administered SBI-46 in xenograft tumors from 22Rv1 and C4-2B cells. Athymic BALB/c nude mice were subcutaneously injected with 22Rv1 and C4-2B (1 × 10^6^ cells), and when the tumors reached 50–100 mm^3^, either the vehicle or SBI-46 (20 mg/kg b.w.) were given orally for five days/week for four weeks. The oral administration of SBI-46 to mice caused a significant decrease in the volume (Figure 5A,D) and weight of tumors as compared to untreated controls (Figure 5B,E). Immunohistochemistry analysis suggests a lower expression of the proliferation markers Ki67, AR, AR-v7, and PSA in SBI-46-treated mice than in vehicle-treated mice (Figure 5C,F).These experiments indicate that the oral administration of SBI-46 exerts an inhibitory effect on the tumor mass 

## 4. Discussion

The aberrant activation of AR is a primary driver of prostate cancer progression; hence, AR is a primary therapeutic target for this disease [28]. The most promising AR inhibitor, enzalutamide, currently used in the clinic, binds directly to AR, blocks androgen binding, attenuates AR’s nuclear localization, and inhibits androgen signaling [29]. Enzalutamide treatment is initially beneficial (IC50 values C4-2B:1.19 µM [30]; LnCaP:1.89 µM; 22RV1: 1.38 µM) [31]; however, CRPC patients will eventually become refractory to this treatment, resulting in a modest overall survival benefit among PCa patients [32]. However, AR signaling could be targeted in several alternative ways. Recent studies suggested that histone-modifying enzymes, such as HDACs, regulate AR signaling [11,14,15,33,34,35], and several HDACi, through their inhibitory action on androgen signaling, have shown promising therapeutic effects in preclinical models. However, HDACi has shown sub-optimal effects and toxicity in clinical studies in CRPC patients, consequently impeding the clinical translational potential of these agents [16,17,36,37,38].

Several mechanisms have been postulated for the resistance of CRPC to ADT. Specifically, the emergence of AR-SV has been linked to the aberrant activation of AR and is a primary reason for enzalutamide resistance [39]. Furthermore, a small-molecule pharmacological inhibitor, niclosamide, has been shown to inhibit AR variants’ expression, sensitizing resistant PCa tumors to enzalutamide or bicalutamide. This result suggests that AR variants (AR-V7) are essential in the resistance to ADT [40].

In this study, we showed that a newly identified novel antiandrogen-equipped HDACi small molecule (SBI-46) effectively inhibits both epigenetic and AR signaling pathways. This results in the upregulation of acetylated H4 and tubulin in LNCaP cells and the inhibition of both AR full-length and AR-V7 expression in CRPC cells. HDAC6 is a well-known regulator of HSP90, which retains and stabilizes full-length AR in the cytosol [41]. Interestingly, SBI-46 shows potent anti-HDAC activity against HDAC 6. The downregulation of AR and AR-SV results in the growth inhibition of a panel of CRPC cells without causing significant toxicity to normal prostate epithelial cells (RWPE-1) and normal kidney cell lines (HEK-293 and VERO). Additionally, AR-null CRPC (PC-3) cells are resistant to SBI-46, suggesting it specifically targets AR-positive PCa cells. Similar combination approaches by targeting AR and HDAC6 have been reported earlier; however, SBI-46 appears more potent than the reported compound, Zeta55, on CRPC [42].

We also confirmed in all three CRPC cell lines (LNCaP, C4-2B, and 22Rv1) that the SBI-46-induced inhibition of AR-FL and AR-SV expression occurs in a time-dependent manner in PCa. Additionally, the expression of PSA, a direct target of AR, is almost abrogated, suggesting that SBI-46 effectively targets AR signaling in CRPC cells. Previous studies suggested AR-V7 is predominately localized in both the cytoplasm and nucleus [43,44]. A previous report suggested that AR-V7 may heterodimerize with full-length AR; presumably, AR-V7 translocates along with full-length AR during its activation. Our results suggest that AR-V7 is predominantly localized in the nucleus compared to the cytoplasm. Targeting nuclear AR-V7 could be challenging. We were to observe that SBI-46 overcame this challenge by inhibiting the expression of nuclear AR-V7 2 in 22Rv1 cells. Previous studies revealed that blocking AR nuclear localization is a viable approach to inhibiting AR signaling [45] Similarly, in our study, the analysis of the cyto-nuclear fraction suggested that SBI-46 inhibits cytosolic full-length AR expression and abolishes AR shuttling to the nucleus in CRPC cells. Additionally, immunofluorescence analysis in our study also showed the downregulation of the nuclear localization of AR as well as AR-V7 in 22Rv1 cells. Small molecules such as EPPI and CPPI have been shown to inhibit AR’s nuclear localization, inhibiting the growth of AR-positive CRPC cells [46]. Apart from the activation and accumulation of nuclear AR, one of the reasons for the failure of ADT in CRPC patients is due to the synthesis of intratumoral androgen that reactivates AR and promotes AR-mediated CRPC growth [47,48,49]. Hence, we challenged CRPC cells with DHT and investigated if SBI-46 can resist DHT-induced AR signaling. We observed that SBI-46 effectively resisted DHT by blocking the nuclear localization of AR in CRPC cells.

It has been reported that the inhibition of AR-mediated survival signaling by small molecules results in either cell cycle arrest or the induction of apoptosis in CRPC cells [50]. Our studies also observed the induction of pro-apoptotic machinery and concomitant downregulation of pro-survival signaling in SBI-46-treated CRPC cells. Xenograft studies demonstrated that the oral administration of SBI-46 inhibited both C4-2B and 22Rv1 tumors in murine models. Furthermore, immunohistochemistry analysis on tumor samples revealed that SBI-46 severely compromised tumor viability, evidenced by lower expression of the proliferation markers Ki67, AR, AR-v7, and PSA in SBI-46-treated compared to the vehicle-treated mice. Additionally, this in vivo study indicated that SBI-46 was not toxic and that the gross pathological examination in vital organs appeared unaffected. Collectively, these observations validated our in vitro findings on SBI-46 and suggested that targeting AR by genetic and epigenetic approaches is an effective strategy for CRPC.

## 5. Conclusions

We identified a novel AR inhibitor, SBI-46, which significantly inhibited growth and induced apoptosis in CRPC cells without causing significant toxicity to healthy immortalized cells. Furthermore, our in vivo data suggest that SBI-46 is a promising compound that may overcome full-length and AR-SV-mediated resistance to androgen ablation therapy in CRPC. Further analysis may reveal whether SBI-46, in combination with chemotherapy, may enhance sensitivity in CRPC. Other comprehensive preclinical studies are warranted to evaluate the efficacy and toxicity of SBI-46 as a single agent or in combination therapy for treating CRPC.

## Data Availability

The data supporting this study’s findings are available from the corresponding authors upon reasonable request.

## Supplementary Materials

The following supporting information can be downloaded at: https://www.mdpi.com/article/10.3390/cancers15061769/s1, Figure S1: Dose–response curve for AR inhibition activity of SBI-46; Figure S2: Dose–response curve for HDAC1, HDAC6, and HDAC8 inhibition activities of SBI-46; Figure S3: FACS analysis of Annexin V-FITC and PI-stained, SBI-46-treated C4-2B cells; Figure S4: Full pictures of the Western blots.
