# Supplementary figures and images for: Antiandrogen-Equipped Histone Deacetylase Inhibitors Selectively Inhibit Androgen Receptor (AR) and AR-Splice Variant (AR-SV) in Castration-Resistant Prostate Cancer (CRPC)
Source: Cancers (Basel). 2023 Mar 15;15(6):1769. doi: 10.3390/cancers15061769 (PMC10046692; doi:10.3390/cancers15061769)

Figure S1

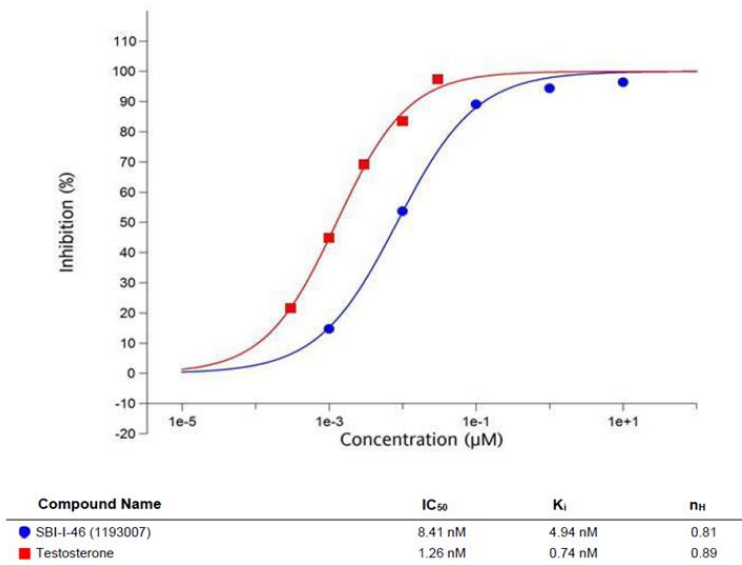

Figure S2

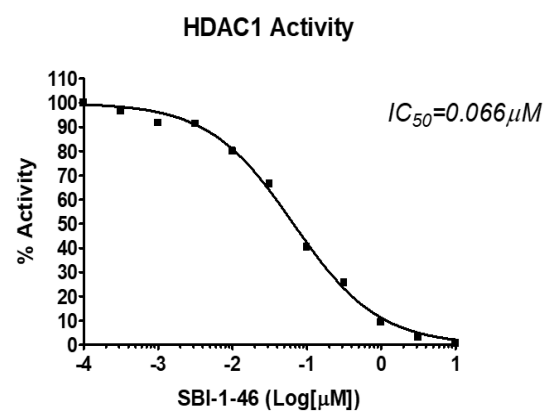

Figure S3

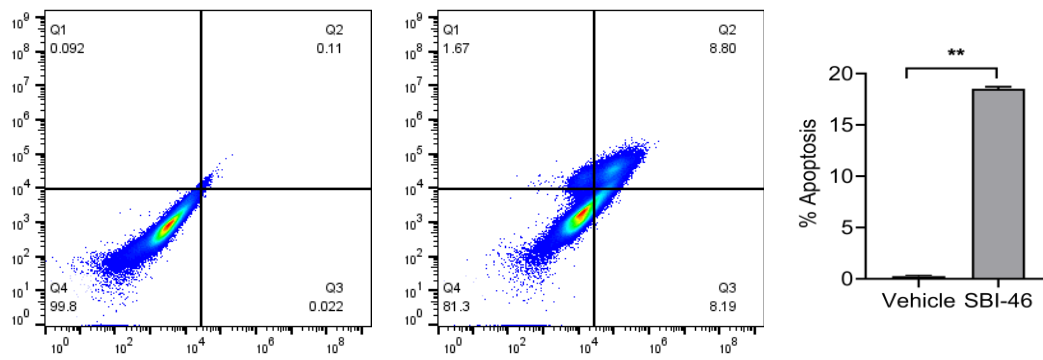

Figure S4

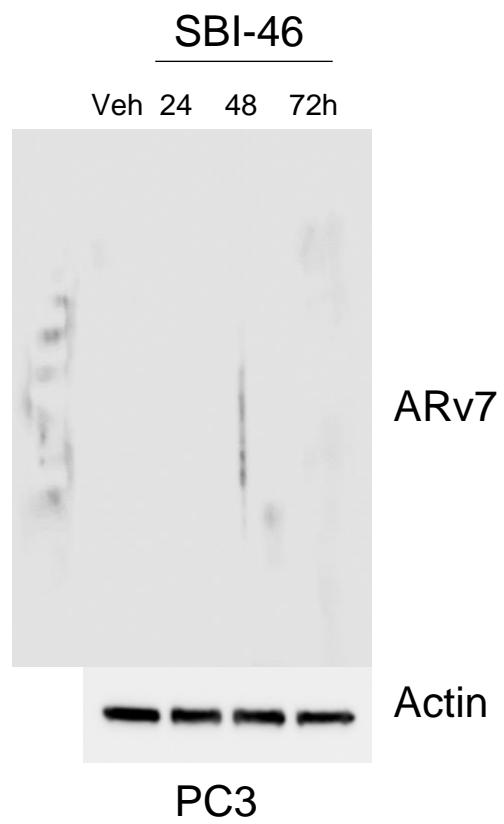

Supplement: Supplementary file 1 [file cancers-15-01769-s001.zip › cancers-2237818-supplementary.pdf]
